# Therapeutic Potential to Modify the Mucus Barrier in Inflammatory Bowel Disease

**DOI:** 10.3390/nu8010044

**Published:** 2016-01-14

**Authors:** Jing Sun, Xiao Shen, Yi Li, Zhen Guo, Weiming Zhu, Lugen Zuo, Jie Zhao, Lili Gu, Jianfeng Gong, Jieshou Li

**Affiliations:** Department of General Surgery, Jinling Hospital, Medical School of Nanjing University, 305 East Zhongshan Road, Nanjing 210002, China; jingdianjing99@126.com (J.S.); dr_shenxiao@126.com (X.S.); dr_liyi@126.com (Y.L.); dr_guozhen@126.com (Z.G.); dr_zuolugen@126.com (L.Z.); dr_zhaojie@126.com (J.Z.); dr_gulili@126.com (L.G.); dr_gongjf@126.com (J.G.); dr_lijieshou@126.com (J.L.)

**Keywords:** mucus barrier, inflammatory bowel disease, bacterial penetration, nutrients, immune

## Abstract

Recently, numerous studies have shown that disruption of the mucus barrier plays an important role in the exacerbation of inflammatory bowel disease, particularly in ulcerative colitis. Alterations in the mucus barrier are well supported by published data and are widely accepted. The use of fluorescence *in situ* hybridization and Carnoy’s fixation has revealed the importance of the mucus barrier in maintaining a mutualistic relationship between host and bacteria. Studies have raised the possibility that modulation of the mucus barrier may provide therapies for the disease, using agents such as short-chain fatty acids, prebiotics and probiotics. This review describes changes in the mucus barrier of patients with inflammatory bowel disease and in animal models of the disease. We also review the involvement of the mucus barrier in the exacerbation of the disease and explore the therapeutic potential of modifying the mucus barrier with short-chain fatty acids, prebiotics, probiotics, fatty acid synthase, H_2_S, neutrophil elastase inhibitor and phophatidyl choline.

## 1. Introduction

Inflammatory bowel disease (IBD), including Crohn’s disease (CD) and ulcerative colitis (UC), is a chronic relapsing disorder characterized by inflammation and mucosal tissue damage of the gastrointestinal tract. It has been suggested that IBD may result from: (1) an imbalance of intestinal microbiota characterized by changes in the composition, quantity and diversity of the microbiota; (2) an altered mucosal barrier structure and function; (3) an aberrant innate immune response; and (4) an imbalanced of T cell responses [1,2].

**Figure 1:**
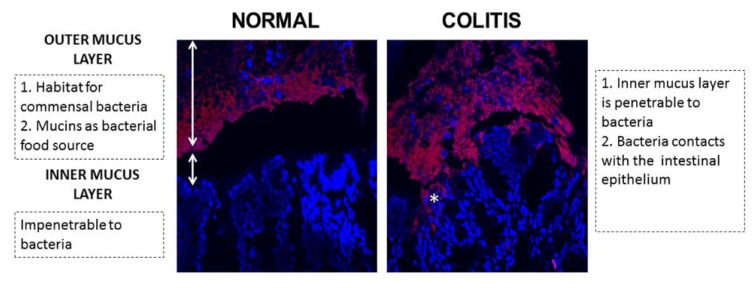
Fluorescence *in situ* hybridization result of normal and colitis colon mucus layers in mice. Red: bacteria; blue: epithelium cells; *: bacteria contacts with the intestinal epithelium.

The primary defence against microbe and pathogen penetration of the lamina propria is the single layer of epithelial cells and its associated protective mucus layer. Increasing evidence suggests that an imbalanced mucus barrier may play an important role in the disease progression of IBD [3,4]. As the first anatomical site of the intestinal barrier, the mucus barrier forms a matrix preventing particles the size of bacteria from penetrating the epithelium. Additionally, as an important part of the innate immune system, the mucus barrier helps to maintain the immune mutualistic relationship between host and bacteria and to reduce the activation of subepithelial lymphocytes [5,6]. In addition, the well-accepted animal models for IBD, dextran sulfate sodium (DSS) induced mice and *Il10*^−/−^ mice, reveal bacterial penetration into the inner mucus layer and epithelial crypt [5,7] (Figure 1), implying dysfunction in the mucus barrier in the exacerbation of IBD. Aberrations in the mucus barrier most likely reduce the ability of the intestinal barrier to cope with bacteria and may contribute to the susceptibility to developing colitis [8].

With the implementation of new technologies, such as fluorescence *in situ* hybridization and Carnoy’s fixation, the composition and function of the mucus barrier between the intestinal microbiota and the intestinal epithelium were able to be investigated more fully. The small intestine has one layer of unattached mucus and directly forms a soluble mucus gel [9]. The mucus thus form a meshed matrix where the diffusion of large components or organisms is slow, and smaller digested nutrients pass more easily and reach the epithelial cells for uptake [10]. The mucus acts as a matrix and antimicrobial products secreted from the epithelial cells can diffuse into this matrix and by this mechanism, limit the contact of microorganism with the cell surface [11]. Colonic mucus consists of two layers: an inner “firmly” adherent mucus layer forms the physical barrier against bacteria, and outer “loose” non-adherent mucus layer generates the preferred habitat for the commensal microbes [12]. Goblet cells secrete mucus into the inner mucus layer through so-called compound exocytosis, and the inner layer transforms into the outer layer. An estimate of the turnover of the inner mucus layer in live murine distal colonic tissue is fast, approximately 1–2 h [13], which is important for maintaining this inner mucus layer free from bacteria [14]. The secretion of mucin from the apical surface is normally constitutive but increases in response to a variety of external stimuli, which helps to reinforce the barrier and flush bacteria from the normally sterile crypts [15]. It was reported that mucin secretion was markedly increased in mice during infection compared to uninfected controls [6]. Goblet cells in the upper crypt do not seem to synthesize enough mucin to meet a constant stimulus because the replenishment of new goblet cells is too slow (longer than 4–5 h) to replace or refill these mucin filled cells [16]. This suggests that continuous stress will limit mucin availability and result in mucus defects [4].

In addition to composition and quantity, the quality of the mucus barrier is also crucial for the function of the barrier. MUC2, as the main secreted mucin in the intestine, is a large and heavily *O*-glycosylated gel-forming mucin that forms enormous polymeric nets by C-terminal dimerization and N-terminal trimerization [12,17]. Mucus quality is associated with correct glycosylation, sialylation and sulfation of mucins [15]. Glycans cover the protein backbone and thus protect the mucin from proteolytic enzymes. The normal degradation of mucin glycans is relatively slow as one monosaccharide is removed at a time. If the glycans become short, the degradation process will be too rapid and not only the outer mucus layer but also the inner can be degraded to allow bacteria to reach the epithelium [18,19]. Gouyer V. *et al.*, reported that delivery of a mucin domain enriched in cysteine residues into the gut lumen can alter the *O*-glycosylation and strengthen the intestinal mucous barrier [20]. In addition, sulfation and sialylation play a role in the resistance of mucin to bacterial degradation. Increased sulfation of mucin was found to yield a gel with a higher viscosity, which is predicted to be more resistant to physical erosion [21].

## 2. Changes in the Mucus Barrier in Inflammatory Bowel Disease

### 2.1. Animal Models

DSS mice, as the most commonly used experimental model of colitis [22], exhibit rapid alterations in the inner mucus layer of the colon that make it permeable to bacteria. Defects in mucosal cell barrier function are related to depletion of the thick viscous mucus layer, goblet cells and mucin, resulting in mucosal inflammation and diarrhea [23]. When 5% DSS was given in the drinking water to mice, bacteria were shown to penetrate the inner mucus layer within 4 h and reach the epithelial cells within 12 h, long before any infiltration of inflammatory cells [24]. In DSS-treated rat, the level of Muc2 and Muc3 increased during the first two days and decreased sharply from day 3, and was consistent with the change of disease activity index (DAI) score, which revealed that mucosal inflammation correlates with MUC2 synthesis [25]. By day 2, goblet cells were filled with mucin (87%) and the adherent mucus layer was thick in the absence of tissue injury or abnormal cellular infiltration. By day 5, intense mucus secretion activity resulted in goblet cells becoming depleted of mucin with a thick non-adherent mucus layer on the surface epithelium. At this time, there was also a sharp increase in disease activity index (DAI). By day 7, few goblet cells were observed at the site of formation of well-developed ulcers, the mucus cap was completely lost and goblet cells in areas adjacent to the ulcers had very little mucin. In particular, though empty, goblet cells was significantly increased compared with controls. By day 9, goblet cells were almost absent at the ulcer site. In the areas adjacent to the ulcers, crypts were damaged, and the few goblet cells contained insignificant amount of mucin.

The *Il10^−/−^* mouse is a well-accepted model of colitis for studying the mucus barrier [26]. *Il10^−/−^* mice display a morphologically stratified inner mucus layer that is still penetrable to bacteria [7]. The thickness of both the inner mucus and the total mucus layer are not decreased, being even thicker *in vivo* and *ex vivo* in *Il10^−/−^* mice. Furthermore, the number of goblet cells and size of the mucus granulae in the goblet cells are not different from those in the wild type mice, which implies an attempt to overcome the mucus barrier defect. These results suggest that changes related to mucus amount and properties of the mucus may be more important than thickness of the mucus in *Il10^−/−^* mice. It was reported that IL-10 acts in an anti-inflammatory way on immune cells, and can directly affect the mucus produced by the goblet cells and the mucus properties [8]. This altered mucus in *Il10^−/−^* mice may be due to IL-10 affecting immune cells and goblet cells. Before week 12, these mice does not develop any sign of colitis or inflammatory sign, but mucus defects already before inflammation developed [7]. Johansson ME *et al*., investigated *Il10^−/−^* mice (male 8–12 weeks) and found that these mice had very mild inflammation and normal thickness and stratified pattern of the inner mucus layer. Interestingly, their inner mucus was also penetrated by bacteria. The mucus quality is further investigated, and was found to be defective in *Il10^−/−^* mice. Mucus from *Il10^−/−^* mice was more easily penetrable compared with controls, which allow bacteria to directly contact with the intestinal epithelium.

### 2.2. Ulcerative Colitis

Previous observations and earlier studies on UC patients showed bacteria directly in contact with the epithelium [7,27,28]. A number of specific changes in the mucus barrier have been reported in UC patients, with both the adherent mucus layer and the whole mucus layer being thinner, more variable and partly denuded, compared to controls [29,30]. Histologic analysis of UC patients often shows depletion of recognizable goblet cells, decreased MUC2 synthesis and decreased MUC2 secretion in the colonic epithelium [27,31]. The mucus layer of UC patients also has reduced mucin glycosylation and shortening of the oligosaccharide side chains of mucin [32,33,34]. In addition, decreased sulfation of mucin, which is associated with decreased viscosity and increased susceptibility to erosion and colonic inflammation, was observed in UC patients [35,36].

It has been shown that in UC patients, the degree of mucosal inflammation correlates significantly with a decrease in MUC2 synthesis [37] and secretion [38], implying that the thickness of the mucus gel is affected by the severity of UC. During active inflammation, the mucus layer thickness is reduced, the goblet cell population is depleted, and individual goblet cells contain less mucin than in healthy controls [30,39]. Theodossi A *et al.*, found that during periods of disease remission both the number and appearance of goblet cells return to normal [40]. In addition, the disease course also influences the mucus barrier. Rectal biopsies of 59 UC patients showed that there was no global change of mucus protection until severe UC. As a consequence of large regions lacking mucus, the mucus layer was less effective due to decreased thickness, a loss of goblet cells and decreased secretory potential [35]. Larsson and colleagues found significant alterations in MUC2 *O*-glycosylation with the most severe patient phenotype and that the glycan pattern reverted to normal when in remission. In active disease, there was a marked shift towards smaller glycans, but the MUC2 glycosylation patterns were similar in controls and UC patients in remission, which indicated that the magnitude of this shift of mucus quality was also significantly correlated with both the degree of inflammation and disease course [41].

### 2.3. Crohn’s Disease

In contrast to UC, the mucus layer is thicker in CD subjects compared with controls. This was mirrored by the yields of mucin obtained from whole-gut lavage, which were low in UC but high in CD [30,42]. In addition, detectable MUC2 protein is increased in CD, irrespective of inflammation. CD, unlike UC, is deep seated, therefore cytokines may initially stimulate mucus secretion, increase the mucus layer thickness, which may explain why MUC2 protein increases in CD patients, but it begins to impair mucus production when the inflammation becomes more extensive [43,44]. The quality of the mucus barrier is also changed, and the oligosaccharide chain length is reduced by 50%, yet sialylation is increased [45,46]. Therefore, increased MUC2 probably does not reflect increased synthesis, but rather decreased post transcriptional sulfation and glycosylation along with altered viscoelastic properties of mucus. Quantity and quality changes of mucus in IBD are shown in Table 1.

**Table 1:** Quantity and quality changes of mucus in irritable bowel disease (IBD).

	Ulcerative Colitis	Crohn’s Disease	Reference
Mucus thickness	Decreased	Increased	[29,30,42]
Goblet cell numbers	Decreased	Unchanged/Increased	[27,30,31,42]
MUC 2 protein	Decreased	Increased	[37,38,43,44]
Glycosylation	Decreased	Unknown	[41]
Sulfation	Decreased	Unchanged	[32,33,34,45,46]
Sialylation	Increased	Increased	[32,33,34,45,46]

## 3. The Role of Mucus Barrier Dysfunction in the Exacerbation of Inflammatory Bowel Disease

### 3.1. Gut Microbiota and the Mucus Barrier

Commensal bacteria lining the mucus surface that maintains gut homeostasis are called biofilms. These benefit the host by digesting substrates inaccessible to host enzymes, modulating immunity, and conferring resistance against transient enteropathogens [47,48].

The relationship between the mucus barrier and the biofilms is dynamic. Although mucins are constitutively secreted, infection of mucosal surfaces can result in a rapid release of stored mucin granules to bolster the barrier and exclude pathogens [49]. The importance of mucus for clearance has been shown in a mouse infected with *Trichuris muris*; this species is closely related to *Trichuris trichiura*, which infects the human colon [50]. Mucus barrier defects allow bacteria to penetrate and reach the epithelia. These defects have been observed in mouse strains with genetic loss or defects in the mucin, MUC2, as well as in molecules that are involved in the formation of the MUC2 mucin polymer [51,52]. MUC2 deficiency leads to exacerbated disease by the attaching and effacing (A/E) pathogen, *Citrobacter rodentium*, characterized by an increased rate of pathogen colonization and an inability to clear pathogen burdens through increased mucus secretion [53]. Thus, mucus barrier dysfunction could influence the effects of bacteria on the colonic mucosa and be instrumental in the development of colitis.

Patients with IBD exhibit a dysbiosis of gut microbiota, characterized by mucus heavily loaded with bacteria in the intestine, some of which adhere to, or even invade, the epithelial surface [54,55]. The abundance of the mucolytic bacterium, *Ruminococcus torques*, which has a strong mucin degrading ability, was increased ~100-fold. In contrast, mucolytic bacteria present in healthy controls, such as *Akkermansia muciniphila*, which has weak mucin degrading abilities, was reduced many fold in macroscopically and histologically normal intestinal epithelium of both CD and UC [56]. In addition, certain enteric pathogens have evolved strategies to circumvent the mucus barriers. Bacteria can secrete not only carbohydrate degrading enzymes but also proteases. Examples are the toxin released from *Bacteroides fragilis* that has been shown to be a proteolytic enzyme [57] and the protease secreted by the oral bacteria, *Porphyromonas gingivalis*, that has been shown to be able to cleave MUC2 [58]. More recently, several *E. coli* family proteases with similar properties have been identified [59].

High levels of sulfate in mucin decrease its susceptibility to bacterial glycosidases and limit the rate and extent of degradation. Therefore, it has been proposed that reduced mucin sulfation might be closely correlated with the increase in bacterial translocation in murine models of gut disease [60]. Approximately 1% of normal colonic bacteria secrete glycosidases and sulphatases capable of degrading mucin oligosaccharides, allowing the enteric microflora to exploit mucin carbohydrates as an energy source [61]. Under these conditions, the mucus barrier remains intact. Some virulent bacteria secrete sulphatases that remove the sulfate ester and thus render the mucin molecule susceptible to degradation by bacterial glycosidases. Bacteria that are capable of cleaving sulfate and utilizing it as a metabolite have been found to be overrepresented in the colitic colon, which may offer an explanation for the reduced sulfated content of mucin in the colitic colon [62]. In UC, there is also increased bacterial sulphatase activity, which may mirror disease activity [63].

### 3.2. The Mucus Barrier and Subepithelial Immune System

*Il10^−/−^* mice kept in a special pathogen free (SPF) environment, which display only minor signs of histological inflammation, still have a mucus layer that can be penetrated by both beads and bacteria. This argues for a link between mucus properties, the immune system and the cytokines produced [64]. As the important element of innate immunity, the mucus barrier is impaired in IBD. Bacterial products and dietary antigens penetrate the mucus layer, cross the epithelium and enter the lamina propria. Most of the immune system resides in the subepithelial compartment, and the antigen-presenting cells (APCs) are ready to take up and present antigens (such as bacteria) to T cells for action or tolerance development [65]. The cytokines from APCs regulate the abnormal differentiation of T cells, which secrete a large number of pro-inflammatory mediators. The active adaptive immune responses are protective for the host in normal conditions, but the response is constantly amplified in IBD [66].

Both host innate and adaptive immunity can regulate the differentiation of goblet cells, the glycosylation of mucins, and the production rates of antimicrobial molecules and cell surface mucins. For many years, it has been assumed that IBD is a T cell-mediated disease [16]. Th1 cells and a novel subset of IL-17-producing CD4+ Th cells, Th17 cells, have more recently been implicated in the pathogenesis of CD [67]. In contrast, UC was reported to be associated with up-regulation of Th2 cells. It was reported that cytokines produced by Th2 cells (such as IL-4, IL-13) in response to parasitic infections, can promote goblet cell hyperplasia and substantially increased mucus production in the intestine [68]. In addition, interferon-γ (IFN-γ) and IL-17, which are classically produced by Th1 cells and Th17 cells in response to intracellular and extracellular pathogens, respectively, affect goblet cells by increasing mucin production [69,70]. T cell cytokines also mediate changes in mucin glycosylation, probably representing an attempt by the host to alter the pattern of glycosylation that fail to prevent infection by a pathogen or parasite [71].

### 3.3. Autophagy and the Mucus Barrier

Autophagy refers to an intracellular pathway involving lysosome-dependent catabolism of proteins and organelles for recycling. Genome-wide association studies of IBD identified susceptible loci containing an autophagy related-gene, *ATG16L1* [72]. Data have also suggested that impaired autophagy might contribute to an increased susceptibility to CD [73].

Conversion of the cytosolic protein LC3-I into phosphatidylethanolamine-conjugated LC3-II on the phagophore surface, catalyzed by autophagy proteins such as Atg7, Atg5 and Atg3, is essential for phagophore expansion and closure of the autophagosome [17]. Loss of autophagy proteins leads to defects in the function of various secretory cell types [74]. It has been reported that *Atg7^−/−^* mice and Atg5 heterozygous mice have impaired mucus secretion from goblet cells [75]. ATG7 is an autophagy-related E1-like enzyme that is essential for two ubiquitination-like reactions (ATG12-conjugation and LC3-lipidation). Researchers took advantage of the specific Cre recombinase expression in colonic epithelial cells in a GlcNAc6ST-2-Cre transgenic mouse model [76] to delete Atg7 in a colonic epithelial cell-specific manner. By using these mutant mice, the function of autophagy was investigated in maintenance of gut commensal microflora and protection against UC-like colitis. Secretion of colonic mucins that function as a mucosal barrier against bacterial invasion was significantly diminished in cKO mice [77].

The NLRP6 inflammasome, a recently described regulator of colonic microbiota composition and bio-geographical distribution, is a critical orchestrator of goblet cell mucin granule exocytosis [75]. Importantly, NLRP6 deficiency leads to abrogation of autophagy in goblet cells and abrogates mucin secretion into the large intestinal lumen, characterized by protruding mucin granules sloughed off into the intestinal lumen rather than fusing into the apical basement membrane and releasing their content. NLRP6-deficient can result in microbial dysbiosis, and intestinal bacteria contacts with the epithelium through destroyed mucus barrier and then results in inflammation. In addition, NLRP6-deficient mice have increased susceptibility to DSS, and the inflammation was more severe compared with controls [75]. Consequently, NLRP6 inflammasome-deficient mice are unable to clear enteric pathogens from the mucosal surface, rendering them highly susceptible to persistent infection, which provides a link between inflammasome activity, autophagy, mucus granule exocytosis, and antimicrobial barrier function [78,79].

## 4. Implications for Clinical Utility

**Figure 2:**
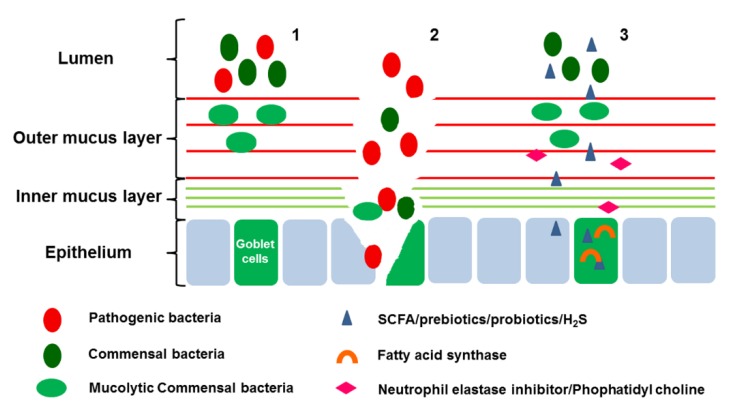
Therapeutic potential of modifying the mucus barrier. (1): Mucins can act as a barrier to both pathogenic and commensal bacteria. Some commensal bacteria are capable of binding to the mucus layer and in do so act as antagonists to the binding of pathogen; (2): When the mucus layer was destroyed, pathogenic bacteria penetrate the mucus layer and bind to the epithelium and exert a negative effect on the host cells; (3) Short-chain fatty acids (SCFA)/prebiotics/probiotics/H_2_S can modulate the bacteria, support nutrition to host cells, and promote the mucus secretion; Fatty acid synthase can also promote the expression of MUC2; Neutrophil elastase inhibitor/Phophatidyl choline can decrease the degradation of mucus layer.

Mucosal healing as a therapeutic aim has become a common endpoint in clinical trials in addition to traditional subjective clinical indices. The mucus barrier, as the first anatomical site of the mucosal barrier, contacts with numerous luminal microbiota and with the submucosal immune system. Disequilibrium of the mucus barrier plays an important role in delaying healing of the damaged tissue of IBD. In addition, changes in the mucus barrier are clearly associated with disease activity and severity, which promotes the potential of the mucus barrier as a target for therapeutics of IBD (Figure 2).

### 4.1. Short-Chain Fatty Acids

SCFAs, mainly acetic acid, propionic acid, and butyrate, are bacterial fermentation products from indigestible dietary components, such as fiber, and range in concentration from 50 to 100 mM in the colonic lumen [80]. There are five bacterial species that characterize dysbiosis in CD, which point towards a lack of butyrate-producing bacteria in the pathogenesis of the disease. Four of these species show a decrease in CD, namely *F prausnitzii*, *Bifidobacterium adolescentis*, *Dialister invisus* and an uncharacterized species of *Clostridium cluster* XIVa, and one, *Ruminococcus gnavus*, shows an increase [81,82]. Sodium butyrate enemas have been used to treat IBD and have obtained beneficial effects both *in vivo* and *in vitro* [83]. Similarly, administration of exogenous butyrate promotes resistance to experimental colitis [84].

As a major SCFA, butyrate is associated with the glycoprotein production of mucus. It is reported to increase colonic mucus synthesis, promote the expression of MUC2 [85], and up-regulate glycoltransferases present in the endoplasmic reticulum [86]. The effect of butyrate in modulating the mucus barrier may explain the therapeutic effect of butyrate in colitis. The mechanism by which butyrate regulates the mucus barrier is not clear. In addition to providing energy to goblet cells, butyrate was shown to be associated with prostaglandin production [85]. Studies of colon cancer have also shown that butyrate can induce autophagy, which plays a key role in both regulating homeostasis of the intestinal mucosa and protecting against colitis through the maintenance of normal gut microflora and mucus secretion [75,77].

### 4.2. Prebiotics and Probiotics

Studies have also reported that prebiotics and probiotics can influence the mucus barrier. Prebiotics are defined as non-digestible, selectively fermented ingredients that induce specific changes in the activity and composition of the gut microbiota, providing benefits to host well-being and health [87]. Addition of soluble dextrin prebiotic fibers to the diet was reported to reduce proinflammatory cytokine secretion and enterocyte injury in male *Il10^−/−^* mice [88]. Importantly, probiotics can directly stimulate mucin gene expression, synthesis, and secretion to protect the host from pathogenic bacterial invasion [89]. It was found that soy protein can suppress the DSS-induced inflammatory stimulation of MUC2 and TNF-α gene expression [90]. In addition to increased MUC2 expression, mucin sialylation and sulfation were also changed after fiber treatment [91].

Probiotics are usually defined as live micro-organism supplements that, when administered in adequate amounts, confer a health benefit on the host by improving the intestinal microbe balance [92]. Specific bacterial strains have been suggested to have a protective activity against IBD. The fermentation products of these bacteria may play an important role in mucoprotection, being an energy source for intestinal epithelial cells and stimulating mucin secretion [93]. Probiotics can modulate EGF receptor (EGFR) signaling, which has been reported to regulate mucin production [94]. p40, a *Lactobacillus rhamnosus* GG-derived soluble protein, was reported to increase mucin production in the colonic epithelium, thus thickening the mucus layer in the colon of wild type mice through transactivation of the EGFR [95]. Interestingly, the number of goblet cells in mice identified by MUC2 immunostaining is not affected by p40 treatment.

### 4.3. Fatty Acid Synthase

Fatty acid synthase (FAS), an insulin-responsive enzyme essential for *de novo* lipogenesis, helps maintain the mucus barrier by regulating MUC2 in a diabetic mouse model [96]. FAS deficiency impaired MUC2 secretion and was associated with decreased goblet cell MUC2. FAS expression is decreased in the ileum and colon of humans with UC [97], and hyperinsulinemia is associated with protection from clinical relapse in patients with IBD [98], which provides evidence linking FAS and human IBD. The mechanism by which FAS maintains the mucus barrier is not clear. Researchers have found that FAS facilitates *S*-palmitoylation of MUC2 at its N terminus, thereby enabling proper mucin secretion and function. Thus, the role of FAS in maintaining intestinal barrier function may explain the pathogenesis of intestinal inflammation in diabetes and other disorders [99].

### 4.4. H_2_S

In the past decade, hydrogen sulfide (H_2_S) has become recognized as an important signaling molecule that influences many aspects of gastrointestinal function. H_2_S promotes mucosal defense, accelerates healing of gastrointestinal ulcers, and promotes resolution of inflammation [100]. Garlic (*Allium sativum*), which is naturally rich in organosulfurs that release H_2_S, has been suggested to have antimicrobial effects *in vitro* on planktonic Gram-negative and Gram-positive bacteria [101,102].

In addition to a therapeutic use during colitis, H_2_S donors could be used to facilitate the correction of microbiota biofilm dysbiosis and mucus layer reconstitution. Mice that are genetically deficient for a key enzyme in H_2_S production, or wild type mice given a pharmacological inhibitor of that enzyme, develop colitis, along with fragmented biofilms and decreased mucus granule production. Therapeutic delivery of H_2_S into the colon reduced inflammation, restored the microbiota biofilm, and increased the production of mucus granules [103], suggesting that H_2_S donors could be exploited as novel therapeutics for IBD.

### 4.5. Neutrophil Elastase Inhibitor

It was reported that increased recruitment and activation of neutrophils and neutrophil elastase (NE) production damages the airway architecture, which leads to progressive lung dysfunction [104]. NE rapidly degrades gel-forming airway mucins in cystic fibrosis (CF) sputum. Recently, Shashi Chillappagari *et al.*, found that KRP-109, a small molecule NE inhibitor, would inhibit CF mucin degradation *in vitro*, and might rescue mucus clearance and reverse airway obstruction. Similar to the lung, the lumen was infiltrated with neutrophils during colitis, and the effect of NE on colitis and goblet cells remains to be studied.

### 4.6. Phophatidyl Choline

In the small intestine, mucus not only facilitates substrate absorption, but also forms a hydrophobic, phosphatidyl choline (PC) enriched, barrier against luminal gut contents. Robert Ehehalt *et al.*, found that PC in the intestinal mucus originates from secretion by ileal and jejunal enterocytes [105]. Within the colon, PC would most likely adhere strongly to the mucosal surface extending into the rectum. Tight mucus of this type may protect the mucosa from microbes and toxins within the colonic lumen. Studies found that topical application of PC preparations protects intestinal mucosa from injury [106] and has been shown to prevent experimentally induced colitis [107]. Whether PC supplementation could be exploited as novel therapeutics for IBD should be further explored.

## 5. Conclusions and Future Work

The mucus layer has long been recognized as an important ingredient in gut protection, but has attracted less attention during the recent decades. In fact, the intestinal mucus is often missing in illustrations depicting gut protection [108]. In our review, we introduce changes that occur in the mucus barrier in IBD and the associated animal models, the role of mucus barrier dysfunction in the exacerbation of IBD, and potential for therapeutics. Thus, we may conclude that there are obvious mucus barrier dysfunctions in IBD, which are associated with the exacerbation of the disease. Currently, some therapeutics focusing on the mucus barrier is available. Future therapies in IBD should aim to strengthen the mucous barrier. Further studies are required to establish an understanding of host microbe interactions within the mucus barrier. The identification of the epitopes on the mucin molecules that anchor microbes could be potential targets for drugs.

It remains to be answered whether alterations in the mucus layer and its bacterial contamination are primary or secondary factors in IBD. The *Il10^−/−^* mice kept in our animal house display only minor histological inflammatory signs, but still have a mucus layer that is penetrated by both beads and bacteria. Better understanding the changes in the mucin barrier in IBD patients could have ramifications for early detection and therapeutic interventions.

## References

[1] Pandolfi F., Cianci R., Pagliari D., Landolfi R., Cammarota G. (2009). Cellular mediators of inflammation: Tregs and TH17 cells in gastrointestinal diseases. Mediat. Inflamm..

[2] Geremia A., Biancheri P., Allan P., Corazza G.R., di Sabatino A. (2014). Innate and adaptive immunity in inflammatory bowel disease. Autoimmun. Rev..

[3] Boltin D., Perets T.T., Vilkin A., Niv Y. (2013). Mucin function in inflammatory bowel disease: An update. J. Clin. Gastroenterol..

[4] Johansson M.E., Sjovall H., Hansson G.C. (2013). The gastrointestinal mucus system in health and disease. Nat. Rev. Gastroenterol. Hepatol..

[5] Chassaing B., Darfeuille-Michaud A. (2011). The commensal microbiota and enteropathogens in the pathogenesis of inflammatory bowel diseases. Gastroenterology.

[6] Bergstrom K.S., Kissoon-Singh V., Gibson D.L., Ma C., Montero M., Sham H.P., Ryz N., Huang T., Velcich A., Finlay B.B. (2010). Muc2 protects against lethal infectious colitis by disassociating pathogenic and commensal bacteria from the colonic mucosa. PLoS Pathog..

[7] Johansson M.E., Gustafsson J.K., Holmen-Larsson J., Jabbar K.S., Xia L., Xu H., Ghishan F.K., Carvalho F.A., Gewirtz A.T., Sjovall H. (2014). Bacteria penetrate the normally impenetrable inner colon mucus layer in both murine colitis models and patients with ulcerative colitis. Gut.

[8] Schwerbrock N.M., Makkink M.K., van der Sluis M., Buller H.A., Einerhand A.W., Sartor R.B., Dekker J. (2004). Interleukin 10-deficient mice exhibit defective colonic muc2 synthesis before and after induction of colitis by commensal bacteria. Inflamm. Bowel Dis..

[9] Atuma C., Strugala V., Allen A., Holm L. (2001). The adherent gastrointestinal mucus gel layer: Thickness and physical state *in vivo*. Am. J. Physiol. Gastrointest. Liver Physiol..

[10] Ermund A., Schütte A., Johansson M.E., Gustafsson J.K., Hansson G.C. (2013). Studies of mucus in mouse stomach, small intestine, and colon. I. Gastrointestinal mucus layers have different properties depending on location as well as over the Peyer’s patches. Am. J. Physiol. Gastrointest. Liver Physiol..

[11] Vaishnava S., Yamamoto M., Severson K.M., Ruhn K.A., Yu X., Koren O., Ley R., Wakeland E.K., Hooper L.V. (2011). The antibacterial lectin RegIIIgamma promotes the spatial segregation of microbiota and host in the intestine. Science.

[12] Johansson M.E., Larsson J.M., Hansson G.C. (2011). The two mucus layers of colon are organized by the muc2 mucin, whereas the outer layer is a legislator of host-microbial interactions. Proc. Natl. Acad. Sci. USA.

[13] Johansson M.E. (2012). Fast renewal of the distal colonic mucus layers by the surface goblet cells as measured by *in vivo* labeling of mucin glycoproteins. PLoS ONE.

[14] Johansson M.E., Phillipson M., Petersson J., Velcich A., Holm L., Hansson G.C. (2008). The inner of the two muc2 mucin-dependent mucus layers in colon is devoid of bacteria. Proc. Natl. Acad. Sci. USA.

[15] McGuckin M.A., Linden S.K., Sutton P., Florin T.H. (2011). Mucin dynamics and enteric pathogens. Nat. Rev. Microbiol..

[16] Grootjans J., Hundscheid I.H., Lenaerts K., Boonen B., Renes I.B., Verheyen F.K., Dejong C.H., von Meyenfeldt M.F., Beets G.L., Buurman W.A. (2013). Ischaemia-induced mucus barrier loss and bacterial penetration are rapidly counteracted by increased goblet cell secretory activity in human and rat colon. Gut.

[17] Godl K., Johansson M.E., Lidell M.E., Morgelin M., Karlsson H., Olson F.J., Gum J.R., Kim Y.S., Hansson G.C. (2002). The N terminus of the MUC2 mucin forms trimers that are held together within a trypsin-resistant core fragment. J. Biol. Chem..

[18] An G., Wei B., Xia B., McDaniel J.M., Ju T., Cummings R.D., Braun J., Xia L. (2007). Increased susceptibility to colitis and colorectal tumors in mice lacking core 3-derived *O*-glycans. J. Exp. Med..

[19] Fu J., Wei B., Wen T., Johansson M.E., Liu X., Bradford E., Thomsson K.A., McGee S., Mansour L., Tong M. (2011). Loss of intestinal core 1-derived *O*-glycans causes spontaneous colitis in mice. J. Clin. Investig..

[20] Gouyer V., Dubuquoy L., Robbe-Masselot C., Neut C., Singer E., Plet S., Geboes K., Desreumaux P., Gottrand F., Desseyn J.L. (2015). Delivery of a mucin domain enriched in cysteine residues strengthens the intestinal mucous barrier. Sci. Rep..

[21] Sellers L.A., Allen A., Morris E.R., Ross-Murphy S.B. (1988). Mucus glycoprotein gels. Role of glycoprotein polymeric structure and carbohydrate side-chains in gel-formation. Carbohydr. Res..

[22] Axelsson L.G., Landstrom E., Goldschmidt T.J., Gronberg A., Bylund-Fellenius A.C. (1996). Dextran sulfate sodium (DSS) induced experimental colitis in immunodeficient mice: Effects in CD4(+)-cell depleted, athymic and NK-cell depleted SCID mice. Inflamm. Res..

[23] Clayburgh D.R., Shen L., Turner J.R. (2004). A porous defense: The leaky epithelial barrier in intestinal disease. Lab. Investig..

[24] Johansson M.E., Gustafsson J.K., Sjoberg K.E., Petersson J., Holm L., Sjovall H., Hansson G.C. (2010). Bacteria penetrate the inner mucus layer before inflammation in the dextran sulfate colitis model. PLoS ONE.

[25] Dharmani P., Leung P., Chadee K. (2011). Tumor necrosis factor-α and MUC2 mucin play major roles in disease onset and progression in dextran sodium sulphate-induced colitis. PLoS ONE.

[26] Izcue A., Coombes J.L., Powrie F. (2009). Regulatory lymphocytes and intestinal inflammation. Annu. Rev. Immunol..

[27] Swidsinski A., Loening-Baucke V., Theissig F., Engelhardt H., Bengmark S., Koch S., Lochs H., Dorffel Y. (2007). Comparative study of the intestinal mucus barrier in normal and inflamed colon. Gut.

[28] Machiels K., Joossens M., Sabino J., de Preter V., Arijs I., Eeckhaut V., Ballet V., Claes K., van Immerseel F., Verbeke K. (2014). A decrease of the butyrate-producing species roseburia hominis and faecalibacterium prausnitzii defines dysbiosis in patients with ulcerative colitis. Gut.

[29] Lennon G., Balfe A., Bambury N., Lavelle A., Maguire A., Docherty N.G., Coffey J.C., Winter D.C., Sheahan K., O’Connell P.R. (2014). Correlations between colonic crypt mucin chemotype, inflammatory grade and desulfovibrio species in ulcerative colitis. Colorectal Dis..

[30] Pullan R.D., Thomas G.A., Rhodes M., Newcombe R.G., Williams G.T., Allen A., Rhodes J. (1994). Thickness of adherent mucus gel on colonic mucosa in humans and its relevance to colitis. Gut.

[31] Gersemann M., Becker S., Kubler I., Koslowski M., Wang G., Herrlinger K.R., Griger J., Fritz P., Fellermann K., Schwab M. (2009). Differences in goblet cell differentiation between crohn’s disease and ulcerative colitis. Differentiation.

[32] Heazlewood C.K., Cook M.C., Eri R., Price G.R., Tauro S.B., Taupin D., Thornton D.J., Png C.W., Crockford T.L., Cornall R.J. (2008). Aberrant mucin assembly in mice causes endoplasmic reticulum stress and spontaneous inflammation resembling ulcerative colitis. PLoS Med..

[33] Willing B.P., Dicksved J., Halfvarson J., Andersson A.F., Lucio M., Zheng Z., Jarnerot G., Tysk C., Jansson J.K., Engstrand L. (2010). A pyrosequencing study in twins shows that gastrointestinal microbial profiles vary with inflammatory bowel disease phenotypes. Gastroenterology.

[34] Croix J.A., Carbonero F., Nava G.M., Russell M., Greenberg E., Gaskins H.R. (2011). On the relationship between sialomucin and sulfomucin expression and hydrogenotrophic microbes in the human colonic mucosa. PLoS ONE.

[35] Strugala V., Dettmar P.W., Pearson J.P. (2008). Thickness and continuity of the adherent colonic mucus barrier in active and quiescent ulcerative colitis and crohn’s disease. Int. J. Clin. Pract..

[36] Gouyer V., Gottrand F., Desseyn J.L. (2011). The extraordinarily complex but highly structured organization of intestinal mucus-gel unveiled in multicolor images. PLoS ONE.

[37] Tytgat K.M., van der Wal J.W., Einerhand A.W., Buller H.A., Dekker J. (1996). Quantitative analysis of MUC2 synthesis in ulcerative colitis. Biochem. Biophys. Res. Commun..

[38] Van Klinken B.J., van der Wal J.W., Einerhand A.W., Buller H.A., Dekker J. (1999). Sulphation and secretion of the predominant secretory human colonic mucin muc2 in ulcerative colitis. Gut.

[39] McCormick D.A., Horton L.W., Mee A.S. (1990). Mucin depletion in inflammatory bowel disease. J. Clin. Pathol..

[40] Theodossi A., Spiegelhalter D.J., Jass J., Firth J., Dixon M., Leader M., Levison D.A., Lindley R., Filipe I., Price A. (1994). Observer variation and discriminatory value of biopsy features in inflammatory bowel disease. Gut.

[41] Larsson J.M., Karlsson H., Crespo J.G., Johansson M.E., Eklund L., Sjovall H., Hansson G.C. (2011). Altered *O*-glycosylation profile of MUC2 mucin occurs in active ulcerative colitis and is associated with increased inflammation. Inflamm. Bowel Dis..

[42] Saitoh H., Takagaki K., Nakamura T., Munakata A., Yoshida Y., Endo M. (1996). Characterization of mucin in whole-gut lavage fluid obtained from patients with inflammatory bowel disease. Dig. Dis. Sci..

[43] Smirnova M.G., Birchall J.P., Pearson J.P. (2000). Tnf-alpha in the regulation of muc5ac secretion: Some aspects of cytokine-induced mucin hypersecretion on the *in vitro* model. Cytokine.

[44] Smirnova M.G., Kiselev S.L., Birchall J.P., Pearson J.P. (2001). Up-regulation of mucin secretion in HT29-MTX cells by the pro-inflammatory cytokines tumor necrosis factor-α and interleukin-6. Eur. Cytokine Netw..

[45] Raouf A.H., Tsai H.H., Parker N., Hoffman J., Walker R.J., Rhodes J.M. (1992). Sulphation of colonic and rectal mucin in inflammatory bowel disease: Reduced sulphation of rectal mucus in ulcerative colitis. Clin. Sci..

[46] Parker N., Tsai H.H., Ryder S.D., Raouf A.H., Rhodes J.M. (1995). Increased rate of sialylation of colonic mucin by cultured ulcerative colitis mucosal explants. Digestion.

[47] Kostic A.D., Xavier R.J., Gevers D. (2014). The microbiome in inflammatory bowel disease: Current status and the future ahead. Gastroenterology.

[48] Sommer F., Backhed F. (2013). The gut microbiota—Masters of host development and physiology. Nat. Rev. Microbiol..

[49] Thornton D.J., Rousseau K., McGuckin M.A. (2008). Structure and function of the polymeric mucins in airways mucus. Annu. Rev. Physiol..

[50] Hasnain S.Z., Evans C.M., Roy M., Gallagher A.L., Kindrachuk K.N., Barron L., Dickey B.F., Wilson M.S., Wynn T.A., Grencis R.K. (2011). Muc5ac: A critical component mediating the rejection of enteric nematodes. J. Exp. Med..

[51] Kankainen M., Paulin L., Tynkkynen S., von Ossowski I., Reunanen J., Partanen P., Satokari R., Vesterlund S., Hendrickx A.P., Lebeer S. (2009). Comparative genomic analysis of lactobacillus rhamnosus GG reveals pili containing a human-mucus binding protein. Proc. Natl. Acad. Sci. USA.

[52] Laubitz D., Larmonier C.B., Bai A., Midura-Kiela M.T., Lipko M.A., Thurston R.D., Kiela P.R., Ghishan F.K. (2008). Colonic gene expression profile in NHE3-deficient mice: Evidence for spontaneous distal colitis. Am. J. Physiol. Gastrointest. Liver Physiol..

[53] Bergstrom K.S., Guttman J.A., Rumi M., Ma C., Bouzari S., Khan M.A., Gibson D.L., Vogl A.W., Vallance B.A. (2008). Modulation of intestinal goblet cell function during infection by an attaching and effacing bacterial pathogen. Infect. Immun..

[54] Duboc H., Rajca S., Rainteau D., Benarous D., Maubert M.A., Quervain E., Thomas G., Barbu V., Humbert L., Despras G. (2013). Connecting dysbiosis, bile-acid dysmetabolism and gut inflammation in inflammatory bowel diseases. Gut.

[55] Walker A.W., Sanderson J.D., Churcher C., Parkes G.C., Hudspith B.N., Rayment N., Brostoff J., Parkhill J., Dougan G., Petrovska L. (2011). High-throughput clone library analysis of the mucosa-associated microbiota reveals dysbiosis and differences between inflamed and non-inflamed regions of the intestine in inflammatory bowel disease. BMC Microbiol..

[56] Png C.W., Linden S.K., Gilshenan K.S., Zoetendal E.G., McSweeney C.S., Sly L.I., McGuckin M.A., Florin T.H. (2010). Mucolytic bacteria with increased prevalence in ibd mucosa augment *in vitro* utilization of mucin by other bacteria. Am. J. Gastroenterol..

[57] Rhee K.J., Wu S., Wu X., Huso D.L., Karim B., Franco A.A., Rabizadeh S., Golub J.E., Mathews L.E., Shin J. (2009). Induction of persistent colitis by a human commensal, enterotoxigenic bacteroides fragilis, in wild-type c57bl/6 mice. Infect. Immun..

[58] Van der Post S., Subramani D.B., Backstrom M., Johansson M.E., Vester-Christensen M.B., Mandel U., Bennett E.P., Clausen H., Dahlen G., Sroka A. (2013). Site-specific *O*-glycosylation on the muc2 mucin protein inhibits cleavage by the porphyromonas gingivalis secreted cysteine protease (RgpB). J. Biol. Chem..

[59] Pelaseyed T., Bergstrom J.H., Gustafsson J.K., Ermund A., Birchenough G.M., Schutte A., van der Post S., Svensson F., Rodriguez-Pineiro A.M., Nystrom E.E. (2014). The mucus and mucins of the goblet cells and enterocytes provide the first defense line of the gastrointestinal tract and interact with the immune system. Immunol. Rev..

[60] Dawson P.A., Huxley S., Gardiner B., Tran T., McAuley J.L., Grimmond S., McGuckin M.A., Markovich D. (2009). Reduced mucin sulfonation and impaired intestinal barrier function in the hyposulfataemic nas1 null mouse. Gut.

[61] Hoskins L.C., Boulding E.T., Gerken T.A., Harouny V.R., Kriaris M.S. (1992). Mucin Glycoprotein Degradation by Mucin Oligosaccharide-degrading Strains of Human Fecal Fecal Bacteria. Characterisation of Saccharide Cleavage Products and their Potential Role in Nutritional Support of Larger Faecal Bacterial Populations. Microb. Ecol. Health Dis..

[62] Rowan F., Docherty N.G., Murphy M., Murphy B., Calvin Coffey J., O’Connell P.R. (2010). Desulfovibrio bacterial species are increased in ulcerative colitis. Dis. Colon Rectum.

[63] Tsai H.H., Dwarakanath A.D., Hart C.A., Milton J.D., Rhodes J.M. (1995). Increased faecal mucin sulphatase activity in ulcerative colitis: A potential target for treatment. Gut.

[64] Ambort D., Johansson M.E., Gustafsson J.K., Nilsson H.E., Ermund A., Johansson B.R., Koeck P.J., Hebert H., Hansson G.C. (2012). Calcium and ph-dependent packing and release of the gel-forming muc2 mucin. Proc. Natl. Acad. Sci. USA.

[65] Barnes M.J., Powrie F. (2009). Regulatory t cells reinforce intestinal homeostasis. Immunity.

[66] Campieri M., Gionchetti P. (2001). Bacteria as the cause of ulcerative colitis. Gut.

[67] Grootjans J., Hundscheid I.H., Buurman W.A. (2013). Goblet cell compound exocytosis in the defense against bacterial invasion in the colon exposed to ischemia-reperfusion. Gut Microbes.

[68] Whittaker L., Niu N., Temann U.A., Stoddard A., Flavell R.A., Ray A., Homer R.J., Cohn L. (2002). Interleukin-13 mediates a fundamental pathway for airway epithelial mucus induced by cd4 t cells and interleukin-9. Am. J. Respir. Cell Mol. Biol..

[69] Sugimoto K., Ogawa A., Mizoguchi E., Shimomura Y., Andoh A., Bhan A.K., Blumberg R.S., Xavier R.J., Mizoguchi A. (2008). Il-22 ameliorates intestinal inflammation in a mouse model of ulcerative colitis. J. Clin. Investig..

[70] Andrianifahanana M., Singh A.P., Nemos C., Ponnusamy M.P., Moniaux N., Mehta P.P., Varshney G.C., Batra S.K. (2007). Ifn-gamma-induced expression of muc4 in pancreatic cancer cells is mediated by stat-1 upregulation: A novel mechanism for ifn-gamma response. Oncogene.

[71] Takeda K., Hashimoto K., Uchikawa R., Tegoshi T., Yamada M., Arizono N. (2010). Direct effects of il-4/il-13 and the nematode nippostrongylus brasiliensis on intestinal epithelial cells *in vitro*. Parasite Immunol..

[72] Rioux J.D., Xavier R.J., Taylor K.D., Silverberg M.S., Goyette P., Huett A., Green T., Kuballa P., Barmada M.M., Datta L.W. (2007). Genome-wide association study identifies new susceptibility loci for crohn disease and implicates autophagy in disease pathogenesis. Nat. Genet..

[73] Deretic V., Levine B. (2009). Autophagy, immunity, and microbial adaptations. Cell Host Microbe.

[74] DeSelm C.J., Miller B.C., Zou W., Beatty W.L., van Meel E., Takahata Y., Klumperman J., Tooze S.A., Teitelbaum S.L., Virgin H.W. (2011). Autophagy proteins regulate the secretory component of osteoclastic bone resorption. Dev. Cell.

[75] Wlodarska M., Thaiss C.A., Nowarski R., Henao-Mejia J., Zhang J.P., Brown E.M., Frankel G., Levy M., Katz M.N., Philbrick W.M. (2014). Nlrp6 inflammasome orchestrates the colonic host-microbial interface by regulating goblet cell mucus secretion. Cell.

[76] Kawashima H., Hirakawa J., Tobisawa Y., Fukuda M., Saga Y. Conditional gene targeting in mouse high endothelial venules. J. Immunol..

[77] Tsuboi K., Nishitani M., Takakura A., Imai Y., Komatsu M., Kawashima H. (2015). Autophagy protects against colitis by the maintenance of normal gut microflora and secretion of mucus. J. Biol. Chem..

[78] Choi A.M., Ryter S.W., Levine B. (2013). Autophagy in human health and disease. N. Engl. J. Med..

[79] Normand S., Delanoye-Crespin A., Bressenot A., Huot L., Grandjean T., Peyrin-Biroulet L., Lemoine Y., Hot D., Chamaillard M. (2011). Nod-like receptor pyrin domain-containing protein 6 (NLRP6) controls epithelial self-renewal and colorectal carcinogenesis upon injury. Proc. Natl. Acad. Sci. USA.

[80] Ley R.E., Peterson D.A., Gordon J.I. (2006). Ecological and evolutionary forces shaping microbial diversity in the human intestine. Cell.

[81] Joossens M., Huys G., Cnockaert M., de Preter V., Verbeke K., Rutgeerts P., Vandamme P., Vermeire S. (2011). Dysbiosis of the faecal microbiota in patients with crohn’s disease and their unaffected relatives. Gut.

[82] Morgan X.C., Tickle T.L., Sokol H., Gevers D., Devaney K.L., Ward D.V., Reyes J.A., Shah S.A., LeLeiko N., Snapper S.B. (2012). Dysfunction of the intestinal microbiome in inflammatory bowel disease and treatment. Genome Biol..

[83] Tarrerias A.L., Millecamps M., Alloui A., Beaughard C., Kemeny J.L., Bourdu S., Bommelaer G., Eschalier A., Dapoigny M., Ardid D. (2002). Short-chain fatty acid enemas fail to decrease colonic hypersensitivity and inflammation in tnbs-induced colonic inflammation in rats. Pain.

[84] Cresci G., Nagy L.E., Ganapathy V. (2013). Lactobacillus GG and tributyrin supplementation reduce antibiotic-induced intestinal injury. JPEN J. Parenter. Enter. Nutr..

[85] Willemsen L.E., Koetsier M.A., van Deventer S.J., van Tol E.A. (2003). Short chain fatty acids stimulate epithelial mucin 2 expression through differential effects on prostaglandin e(1) and e(2) production by intestinal myofibroblasts. Gut.

[86] Paulson J.C., Colley K.J. (1989). Glycosyltransferases. Structure, localization, and control of cell type-specific glycosylation. J. Biol. Chem..

[87] Roberfroid M. (2007). Prebiotics: The concept revisited. J. Nutr..

[88] Valcheva R., Hotte N., Gillevet P., Sikaroodi M., Thiessen A., Madsen K.L. (2015). Soluble dextrin fibers alter the intestinal microbiota and reduce proinflammatory cytokine secretion in male il-10-deficient mice. J. Nutr..

[89] Khailova L., Dvorak K., Arganbright K.M., Halpern M.D., Kinouchi T., Yajima M., Dvorak B. (2009). Bifidobacterium bifidum improves intestinal integrity in a rat model of necrotizing enterocolitis. Am. J. Physiol. Gastrointest. Liver Physiol..

[90] Jiang H., Przybyszewski J., Mitra D., Becker C., Brehm-Stecher B., Tentinger A., MacDonald R.S. (2011). Soy protein diet, but not lactobacillus rhamnosus gg, decreases mucin-1, trefoil factor-3, and tumor necrosis factor-α in colon of dextran sodium sulfate-treated c57bl/6 mice. J. Nutr..

[91] Hino S., Takemura N., Sonoyama K., Morita A., Kawagishi H., Aoe S., Morita T. (2012). Small intestinal goblet cell proliferation induced by ingestion of soluble and insoluble dietary fiber is characterized by an increase in sialylated mucins in rats. J. Nutr..

[92] Ishibashi N., Yamazaki S. (2001). Probiotics and safety. Am. J. Clin. Nutr..

[93] Shimotoyodome A., Meguro S., Hase T., Tokimitsu I., Sakata T. (2000). Short chain fatty acids but not lactate or succinate stimulate mucus release in the rat colon. Comp. Biochem. Physiol. A Mol. Integr. Physiol..

[94] Nadel J.A. (2001). Role of epidermal growth factor receptor activation in regulating mucin synthesis. Respir. Res..

[95] Wang L., Cao H., Liu L., Wang B., Walker W.A., Acra S.A., Yan F. (2014). Activation of epidermal growth factor receptor mediates mucin production stimulated by p40, a *lactobacillus rhamnosus* GG-derived protein. J. Biol. Chem..

[96] Carvalho F.A., Koren O., Goodrich J.K., Johansson M.E., Nalbantoglu I., Aitken J.D., Su Y., Chassaing B., Walters W.A., Gonzalez A. (2012). Transient inability to manage proteobacteria promotes chronic gut inflammation in TLR5-deficient mice. Cell Host Microbe.

[97] Heimerl S., Moehle C., Zahn A., Boettcher A., Stremmel W., Langmann T., Schmitz G. (2006). Alterations in intestinal fatty acid metabolism in inflammatory bowel disease. Biochim. Biophys. Acta.

[98] Valentini L., Wirth E.K., Schweizer U., Hengstermann S., Schaper L., Koernicke T., Dietz E., Norman K., Buning C., Winklhofer-Roob B.M. (2009). Circulating adipokines and the protective effects of hyperinsulinemia in inflammatory bowel disease. Nutrition.

[99] Bosi E., Molteni L., Radaelli M.G., Folini L., Fermo I., Bazzigaluppi E., Piemonti L., Pastore M.R., Paroni R. (2006). Increased intestinal permeability precedes clinical onset of type 1 diabetes. Diabetologia.

[100] Wallace J.L., Vong L., McKnight W., Dicay M., Martin G.R. (2009). Endogenous and exogenous hydrogen sulfide promotes resolution of colitis in rats. Gastroenterology.

[101] Feng S., Eucker T.P., Holly M.K., Konkel M.E., Lu X., Wang S. (2014). Investigating the responses of cronobacter sakazakii to garlic-drived organosulfur compounds: A systematic study of pathogenic-bacterium injury by use of high-throughput whole-transcriptome sequencing and confocal micro-raman spectroscopy. Appl. Environ. Microbiol..

[102] Ross Z.M., O’Gara E.A., Hill D.J., Sleightholme H.V., Maslin D.J. (2001). Antimicrobial properties of garlic oil against human enteric bacteria: Evaluation of methodologies and comparisons with garlic oil sulfides and garlic powder. Appl. Environ. Microbiol..

[103] Motta J.P., Flannigan K.L., Agbor T.A., Beatty J.K., Blackler R.W., Workentine M.L., da Silva G.J., Wang R., Buret A.G., Wallace J.L. (2015). Hydrogen sulfide protects from colitis and restores intestinal microbiota biofilm and mucus production. Inflamm. Bowel Dis..

[104] Chillappagari S., Müller C., Mahavadi P., Guenther A., Nährlich L., Rosenblum J., Rubin B.K., Henke M.O. (2015). A small molecule neutrophil elastase inhibitor, KRP-109, inhibits cystic fibrosis mucin degradation. J. Cyst. Fibros..

[105] Ehehalt R., Jochims C., Lehmann W.D., Erben G., Staffer S., Reininger C., Stremmel W. (2004). Evidence of luminal phosphatidylcholine secretion in rat ileum. Biochim. BIophys. Acta.

[106] Mourelle M., Guarner F., Malagelada J.R. (1996). Polyunsaturated phosphatidylcholine prevents stricture formation in a rat model of colitis. Gastroenterology.

[107] Fabia R., Ar’Rajab A., Willén R., Andersson R., Ahrén B., Larsson K., Bengmark S. (1992). Effects of phosphatidylcholine and phosphatidylinositol on acetic-acid-induced colitis in the rat. Digestion.

[108] Artis D. (2008). Epithelial-cell recognition of commensal bacteria and maintenance of immune homeostasis in the gut. Nat. Rev. Immunol..

